# Two Cases of Abdominal Obstruction and Tendency to Inflammation, Cured by Spirits of Turpentine

**Published:** 1816-11

**Authors:** John Brennan


*For the London Medical and Physical Journal.*

### *Two Cases of Abdominal Obstruction and Tendency to Inflammation, cured by Spirits of Turpentine, under the management of* JAMES GREEN, Esq. M.D. of Drogheda. Communicated by Dr. BRENAN, with Dr. Green's Letter to him on the subject.

*To John Brenan, Esq. M.D.* 25, *French-street, Dublin.*

MY DEAR SIR,

LONG and severe illness in my family must plead my apology for not having answered your very kind letter, and returning you my sincere thanks for your pamphlet. Be assured it gives me not a little satisfaction to find that the merit of your discovery is likely to overcome the strong prejudice and illiberality it at first met with. Inclosed I send you the cases I mentioned to you; and, although they are not puerperal, they, in some measure, prove the good effect of turpentine in abdominal obstruction and tendency to inflammation. I have only to say, that I was induced to make use of this medicine from reading your valuable pamphlet on Ol. Terebinth., and finding that you had made use of it with success, as I never either read or heard of its being given in such cases before.

I have the honour to be, dear Sir,

Your's very sincerely,

*Drogheda; August* 29, 1815. JAMES GREEN.

---

CASE I.—On the 18th of April, 1813, I was sent for to a Mrs. B——, at Balbriggan, whom I had attended in her lying-in about six weeks before. She had a very good time, recovered well, and was nursing her infant. When I came, I found she had neglected the state of her bowels very much, and had no alvine evacuation for three days, notwithstanding she had taken castor oil, salts, rhubarb, and sal polychrest, and several enemas had been injected for the last twelve hours. She suffered very great pain in the abdomen, domen, had constant thirst, and her stomach rejected all her drink. Her pulse was up to 120. I gave her a bolus containing six grains of calomel and as many of scammony, which she threw off her stomach in about ten minutes after she had taken it. I then gave her a solution of Epsom salts, with infusion of roses, which her stomach also rejected. An injection was now thrown up, with two ounces of olive oil, a large table spoonful of common salt, and about an ounce of *oil of turpentine*, which she retained for about half an hour, when it came away without any stool.

On the morning of the 19th, I gave her a large table-spoonful of the oil of turpentine, in about three ounces of water, which remained on her stomach for about an hour, when it was thrown up. I directed that she should be kept quiet in bed, as her very excruciating pain occasioned her to writhe, and change her posture every ten minutes. She was also desired to take no drink for an hour, at the end of which time I repeated the oil of turpentine, to be taken in the same manner as before, which rested on her stomach. She had two hours' remission of pain, and slept for the first time since her illness. The barley-water now lay on her stomach, in consequence of which I resumed the boluses and injections, and, in about four hours, gave a desert-spoonful of the turpentine. About six in the morning she had a copious evacuation, with which she passed a considerable quantity of hardened fæces. She had several stools in the course of the evening and night, and, in a few days, was restored to perfect health.

Case II.—On the 20th of December, 1814, I was called to see Mr. Sherrard, of Morlay, in the County of Louth, who had been taken ill two days before with pain in the region of the liver, and with constipation. He was in the habit of taking, from time to time, pills containing calomel and scammony, some of which he had taken the two nights previous to my visit, but they had no effect.

On my inquiring, he told me that he had found it difficult to keep his bowels free for the last fortnight, and, for the last six hours, the pain in the region of the liver was considerably increased. I immediately took about sixteen ounces of blood from his arm, and gave him a solution of vitriolated magnesia in an infusion of senna, with tincture of jalap. He took about eight ounces of this mixture, in four divided doses, at the interval of an hour, without producing any evacuation. He had likewise strong purgative injections every two hours, which he retained for some time, when they returned unchanged.

Early in the morning of the 21st (the fourth day of his illness), ness), I gave him eight grains of calomel and fifteen of rhubarb, formed into a bolus with twenty grains of electuary of scammony, which was repeated every fourth hour, for three doses; and applied a large blister over the abdomen. The bolusses were as unsuccessful as any of his former medicines. The pain in the region of the liver was very severe, and extended over the abdomen, which became tense and puffed. This induced me to repeat the blood-letting, when I found the blood highly buffed, though the former had not been so. His pulse were hurried, and never under 110 pulsations in the minute, frequently more. His stomach now rejected every thing he took, and whatever he drank was not retained more than a few moments. He had also an unquenchable thirst. On the morning of the 22d I gave him a table-spoonful of the oleum terebinthinæ, in a glass of water. This was given imprudently, as he had taken a large draught of barley-water before, which he threw up with the ol. tereb. I continued the injections, with the addition of an ounce of oil of turpentine, rubbed with the yolk of an egg, and repeated them every second or third hour. All these were returned unchanged. About four o'clock in the afternoon of the 22d, I repeated the draught, with the oil of turpentine, after which I observed that the next drink he took remained on his stomach. I thought this a favourable opportunity for resuming his purgative medicines, and therefore gave him half an ounce of Epsom salts in a glass of water, and repeated it every second hour. These draughts likewise continued on his stomach, as did the calomel boluses. I now found that the oil of turpentine had produced an immoderate flow of urine, which, in a few hours, terminated in strangury. I am not able to determine whether this arose from the cantharides or the turpentine, but am inclined to think it was from the latter, as the urine was highly impregnated with it; however he had one small motion. I continued the injections and omitted the turpentine, and likewise continued the magnesia vitriolata, which, in a few hours after, produced several copious motions, with several hard lumps, very black and fætid.

There was a circumstance that I have still to remark, which was, that the day after his bowels were opened, and after he had several motions, he sent down stairs for me to inform me that he thought the medicines I had given him were now passing off, as the stool he had just passed smelled strongly of turpentine.

